# The cracking of Scots pine (*Pinus sylvestris*) cones

**DOI:** 10.3389/fpls.2022.982756

**Published:** 2022-10-18

**Authors:** Martin Horstmann, Hannah Buchheit, Thomas Speck, Simon Poppinga

**Affiliations:** ^1^ Botanic Garden, Plant Biomechanics Group, University of Freiburg, Freiburg im Breisgau, Germany; ^2^ Department of Animal Ecology, Evolution and Biodiversity, Ruhr-University Bochum, Bochum, Germany; ^3^ Freiburg Materials Research Center and Institute for Macromolecular Chemistry, University of Freiburg, Freiburg im Breisgau, Germany; ^4^ Cluster of Excellence livMatS, University of Freiburg, Freiburg im Breisgau, Germany; ^5^ Department of Biology, Botanical Garden, Technical University of Darmstadt, Darmstadt, Germany

**Keywords:** hygroscopy, initial opening, pine cone, plant movement, seed release

## Abstract

Pine cones show functionally highly resilient, hygroscopically actuated opening and closing movements, which are repeatable and function even in millions of years old, coalified cones. Although the functional morphology and biomechanics behind the individual seed scale motions are well understood, the initial opening of the cone, which is often accompanied by an audible cracking noise, is not. We therefore investigated the initial opening events of mature fresh cones of Scots pine (*Pinus sylvestris*) and their subsequent motion patterns. Using high-speed and time lapse videography, 3D digital image correlation techniques, force measurements, thermographic and chemical-rheological resin analyses, we are able to draw a holistic picture of the initial opening process involving the rupture of resin seals and very fast seed scale motion in the millisecond regime. The rapid cone opening was not accompanied by immediate seed release in our experiments and, therefore, cannot be assigned to ballistochory. As the involved passive hydraulic-elastic processes in cracking are very fine-tuned, we hypothesize that they are under tight mechanical-structural control to ensure an ecologically optimized seed release upon environmental conditions suitable for wind dispersal. In this context, we propose an interplay of humidity and temperature to be the external “drivers” for the initial cone opening, in which resin works as a crucial chemical-mechanical latch system.

## Introduction

Plant motions fulfil a multitude of different functions, like the sun tracking of leaves and flowers (Darwin and Darwin, 1880; Gilroy and Masson, 2008) or prey capture movements in carnivorous plants (reviewed by Poppinga et al., 2018; Bauer et al., 2021). Many extraordinary fast plant motions (taking place in the millisecond regime) are involved in seed release processes, as observed in witch-hazel (*Hamamelis mollis*) (Poppinga et al., 2019), touch-me-nots (*Impatiens* spp.) (Deegan, 2012) and wood-sorrel (*Oxalis* spp.) (Li et al., 2020). One of the most famous seed dispersal mechanisms, the hygroscopic opening and closing of pine cones (*Pinus* spp.), is known from the existing literature to be relatively slow and to take place in the minutes or even hours regimes (Allen and Wardrop, 1964; Harlow et al., 1964; Quan et al., 2021).

Whereas Shaw (1914); Harlow et al. (1964), and Dawson et al. (1997) revealed the basic structural basis for hydraulic pine cone actuation, Quan et al. (2021) and Eger et al. (2022) just recently investigated the mechanics facilitating this behavior. In summary, an interplay of four tissue types (sclereids, sclerenchyma, “brown tissue”, and epidermises) with different swelling behaviour, water permeability, and mechanics render the hygro-responsiveness of the pine cone scale possible, leading to the observed repetitive cone motions. However, to our knowledge, the initial opening event of a cone has not been documented from a biomechanical viewpoint in detail until now. A study by Aniszewska et al. (2019) has shown that short microwave irradiation of initially closed Scots pine cones leads to their opening due to the rapid loss of moisture, and that viable seeds can be harvested from them. The process of initial opening is ecologically relevant, as it influences the dispersal of the mostly airborne seeds and, ultimately, the survival rate of seedlings (Winsa, 1995; Przybylski et al., 2021). As *Pinus* is widespread in the Northern Hemisphere, control of cone opening has evolved to adjust to varying environmental conditions based on location and/or wild fire occurrence (Keeley, 2012; Przybylski et al., 2021). In order to understand the parameters affecting opening, we investigated this process exemplary in Scots pine (*Pinus sylvestris*).

*P. sylvestris* flowers in spring and female cones ripen for two seasons and open during the following spring, entailing seed dispersal mainly between March and June (McVean, 1963; Carlisle and Brown, 1968). The initial cone opening processes are accompanied by clearly audible cracks, as also reported from other pine species (Allen and Wardrop, 1964; Wyse et al., 2019a). The cones continue to close (under wet environmental conditions) and to re-open (under dry conditions) afterwards. Cone opening and continuative seed shedding therefore take place under dry conditions when wind dispersal is favored (McVean, 1963; Nathan et al., 1999; Poppinga et al., 2017; Wyse et al., 2019b). At the same time, closed cones also protect seeds from seed-eating animals and from wild fire (Campbell, 1955; Carlisle and Brown, 1968; Summers and Proctor, 1999; Keeley, 2012).

Many pine cones show resin on their surfaces and respective reservoirs have also been identified within the seed scales (Allen and Wardrop, 1964; Roy, 1966; Lin et al., 2002; Bae and Kim, 2020). The resin is anti-microbial, anti-fungal and anti-bacterial and keeps parasites away (Berryman, 1972; Phillips and Croteau, 1999; Knebel et al., 2008; Rissanen et al., 2019). In addition to that, resins (and other wound products of plants) are known to be of interest as e.g., natural glue or additives due to their chemical and mechanical properties (Arrieta et al., 2017; Aldas et al., 2020). These oleoresins consist mainly of terpenes such as alpha- and beta-pinene as well as limonene, the exact composition depending on the pine species. Other constituents may include phellandrene, myrcene, caryophellene and pinocarveol (Dormont, 1997; Yang et al., 2010). Another interesting aspect is the temperature-dependent viscosity of natural resins, changing from solid to a viscous liquid at intermediate temperature ranges (Aldas et al., 2020). As cone cracking occurs only during the initial opening, we speculated a mechanical instability (cf. Skotheim and Mahadevan, 2005) to be at play, probably initiated by breakage of a resin seal acting as a latch.

In order to unravel the physical mechanisms and respective environmental conditions leading to initial cone opening in detail, we used high-speed and time lapse videography of cones under different environmental humidity and temperature regimes, investigated the three-dimensional displacement and strain of cone surfaces during actuation, performed force measurements of individual scales, and determined the thermoplastic behavior of the resin, e.g., the glass-transition temperature. In short, we aimed at understanding how the initial opening of pine cones is tailored for its ecological function of seed release under favorable environmental conditions.

## Methods

### Plant material

Plant material was collected in March and April 2021 in Essen and Freiburg, Germany. Fresh, unopened cones were stored in a closed box in a fridge at 5°C at a relative humidity of 90%. Open cones were stored in an open box at room temperature at 65-70% relative humidity.

### High-speed analyses of cone cracking

We installed two spotlights (1,000 W, Hedler C12, Hedler Systemlicht, Runkel, Germany) and positioned a high-speed camera (NX4-S1, Imaging Solutions GmbH, Eningen unter Achalm, Germany) equipped with a 100 mm lens (Makro-Planar 2/100 ZF, Carl Zeiss AG, Oberkochen, Germany) centrally in front of closed cones at a distance of 1 m. The Motion Studio software (v.2.10.05, IDT Inc. Tallahassee, USA) was used for high-speed data acquisition. A datalogger (EasyLog EL-USB-2-LCD, Lascar Electronics, Wiltshire, UK) recording time, temperature and relative humidity was placed directly above the closed cone. A piece of graph paper allowed for length measurements. A small screw was drilled into the base of the cones axis to allow mounting.

The high-speed videos gained were formatted as image series using Fiji (Schindelin et al., 2012), calibrated using the graph paper as scale, and used to measure the speed of seed scales during their respective initial opening movements. The frame rates for the high-speed videos were 1,000-1,630 frames per second, depending on the resolution and aspect ratio of the videos. Seed scale initial opening velocities and accelerations were measured by tracking easily identifiable points at the scale “tips” (i.e., on the umbos, which are the pronounced protuberances on the scales). In the cases when scales vibrated after opening events, their movements were recorded until full stop. If possible, these data were recorded for several scales per opening event, if more than one scale was affected. Released seeds’ widths and heights were measured using a stereomicroscope (Olympus SZX16, Olympus, Tokyo, Japan) with an attached camera (TSO vidmess, Pulsnitz, Germany).

In a second approach, the already initially opened cones were submersed in tap water at room temperature overnight in order to trigger closing and were investigated in the same way on the next day to check for repeated rapid cone opening.

### 3D digital image correlation analyses of scale deformation and strain during cone opening

With the help of 3D digital image correlation (3D-DIC), we were able to analyze deformation and strain occurring on the surfaces of cones, which allowed examining the development of the cone opening scale by scale. We investigated fresh, unopened cones during their initial opening and in a second approach the same cones during their second opening until the final (maximal) opening state of the cones. For the latter, the cones were closed by submersion in tap water overnight, with excess water being removed with paper towels afterwards. A small screw was drilled into the bases of the cones to allow mounting. We covered the surfaces with chalk spray (3D Laser Scanning Spray, Helling GmbH, Heidgraben Germany). On this whitened surface, a stochastic speckle pattern was carefully applied using black spray paint (Liquitex Professional, carbon black, ColArt, Le Mans, France). Such surface spray painting is required for 3D-DIC measurements and has already proven to function reliably (Correa et al., 2020; Sachse et al., 2020; Durak et al., 2022; Eger et al., 2022). Using a stereo camera setup (PixeLink, PL D685CU, pixel size 4.8 μm) equipped with 100 mm lenses (Makro-Planar 2/100 ZF, Carl Zeiss AG, Oberkochen, Germany), time lapses were recorded for up to 72 hours, covering the whole drying and corresponding movement processes. Deformation and strain were subsequently analyzed with the software Aramis 2016 (GOM GmbH, Braunschweig, Germany), which was also used to calibrate the system. In total, five cones (length = 43.4 mm ± 4.5 mm, width = 20 mm ± 1 mm) were analyzed during their initial as well as their secondary opening events.

### Force measurements

For the measurement of forces generated by individual scales during the cone opening events, we carefully cut off the large central scales. Special care was taken to use only scales with intact basal zones, the main actuating region for hygroscopic motion (Reyssat and Mahadevan, 2009; Eger et al., 2022). Scales were clamped tightly at their base using tweezers and placed beneath a force sensor (static load cell, +/- 100 N, Instron, Darmstadt, Germany). In total, we measured the forces of five wet scales from five cones (initially submersed in tap water for 12 hours) during drying. For comparability, all scales touched the measuring plate centrally and at an angle of about 90° as described in detail in Eger et al. (2022). We additionally determined force generation per gram dry weight. Therefore, the weights of fully soaked and dried scales were measured before and after force measurements using a lab balance (Kern ABT 220-5DM, Balingen, Germany).

### Temperature on cone surfaces during opening

During the high speed measurements of initial opening events we measured the actual cone surface temperatures simultaneously with a thermal camera (testo 885 thermal imager, testo AG, Lenzkirch, Germany) and with an infrared thermometer (UniversalTemp, Robert Bosch Power tools GmbH, Stuttgart, Germany). To verify the accuracy of both devices, we measured the temperature of a lab heat plate (IKA RH digital KT/C, Staufen, Germany), yielding three times very similar results with a difference of 0.3°C. Humidity was recorded with a logger (EasyLog EL-USB-2-LCD, Lascar Electronics, Wiltshire, UK).

In a second, more “natural” approach we exposed cones to direct sunlight on five days in August 2021 in the Botanical Garden of the University of Freiburg. As August 2021 was rather untypically cold and cloudy (Freiburg average temperature in August 2021 was 18.3°C, 2°C below the multi-year average, the maximum temperature was 31.3°C), the conditions on our experimental days (~17°C, ~23°C and ~29°C) are likely to resemble a warm day in April 2021 (Freiburg average temperature 11.2°C, max. 25.3°C, all data by Deutscher Wetterdienst (2021). Closed and already opened cones were placed on a black respectively white cardboard under the open sky, using a 5 cm high, hollow cardboard box as a spacer to the ground. As an additional experiment on the white background, a *Pinus* twig was placed under the cones to simulate the surrounding and background a cone is typically facing. Again, temperatures were recorded simultaneously with the thermal camera and infrared thermometer mentioned above. Cones with an initial surface temperature close to the environmental temperature were exposed to sunlight for at least 15 minutes before the measurements started. For the thermal camera, the environmental temperature, relative humidity and distance to the measured object were adjusted *via* the camera’s software. These necessary data were recorded on the fly with a data logger (EasyLog EL-USB-2-LCD, Lascar Electronics, Wiltshire, UK).

With the infrared camera, only the highest measurable temperature was recorded. We recorded surface temperature of the cones as well as of the black/white cardboards used in the experiments.

In an additional experiment, we tested whether cones open also at lower temperatures when relative humidity is low. We therefore placed three unopened cones in a fridge in a closed plastic tank containing silica beads (Silica Gel Orange, Carl Roth GmbH + Co. KG, Karlsruhe, Germany). With this setup, we achieved conditions of 5°C at 10-20% relative humidity, measured with a data logger. Later on, we placed the cones in a heat cabinet (UN30, Memmert GmbH + Co. KG, Büchenbach, Germany) at 50°C and 25% relative humidity, testing whether the degree of cone opening changes under higher temperatures after an opening in the cold.

### Rheological properties of the resin

The resin was obtained by Soxhlet extraction of cones with chloroform (Fisher Chemicals, Zürich, Swiss). The solvent was then removed under reduced pressure. The resin was not soluble in water. Differential scanning calorimetry (DSC) measurements were carried out on a DSC 204 F1 Phoenix (Netzsch, Selb, Germany) with a heating rate of 10 K/min and two heating cycles in air. Viscosities measured with a rheometer (MARS II, Thermo Fisher Scientific, Waltham, USA) with a plate-plate arrangement (20 mm diameter, 0.2 mm gap) at varying shear rates from 0.1 to 100 1/s (100 steps, logarithmic, 5 s per step, 3 s integration time) averaged over all 100 measurements at different temperatures between 50 and 90°C. The tack was measured on an MCR 702 rheometer (AntonPaar, Graz, Austria) with a plate-plate arrangement (8 mm diameter). The lower plate was preheated to 70°C, the resin was applied, and the upper plate was pressed onto the resin until a gap of 0.2 mm was reached. The measuring temperature was applied and after 5 min the upper plate was lifted with a speed of 5 µm/s and 1 mm/s respectively.

### Statistics

Statistics were carried out using R (R Core Team, 2021). Since data were not-normally distributed, we conducted the non-parametric Wilcoxon-tests for pair-wise comparisons.

## Results

### High-speed analyses of cone cracking

**Figure 1:**
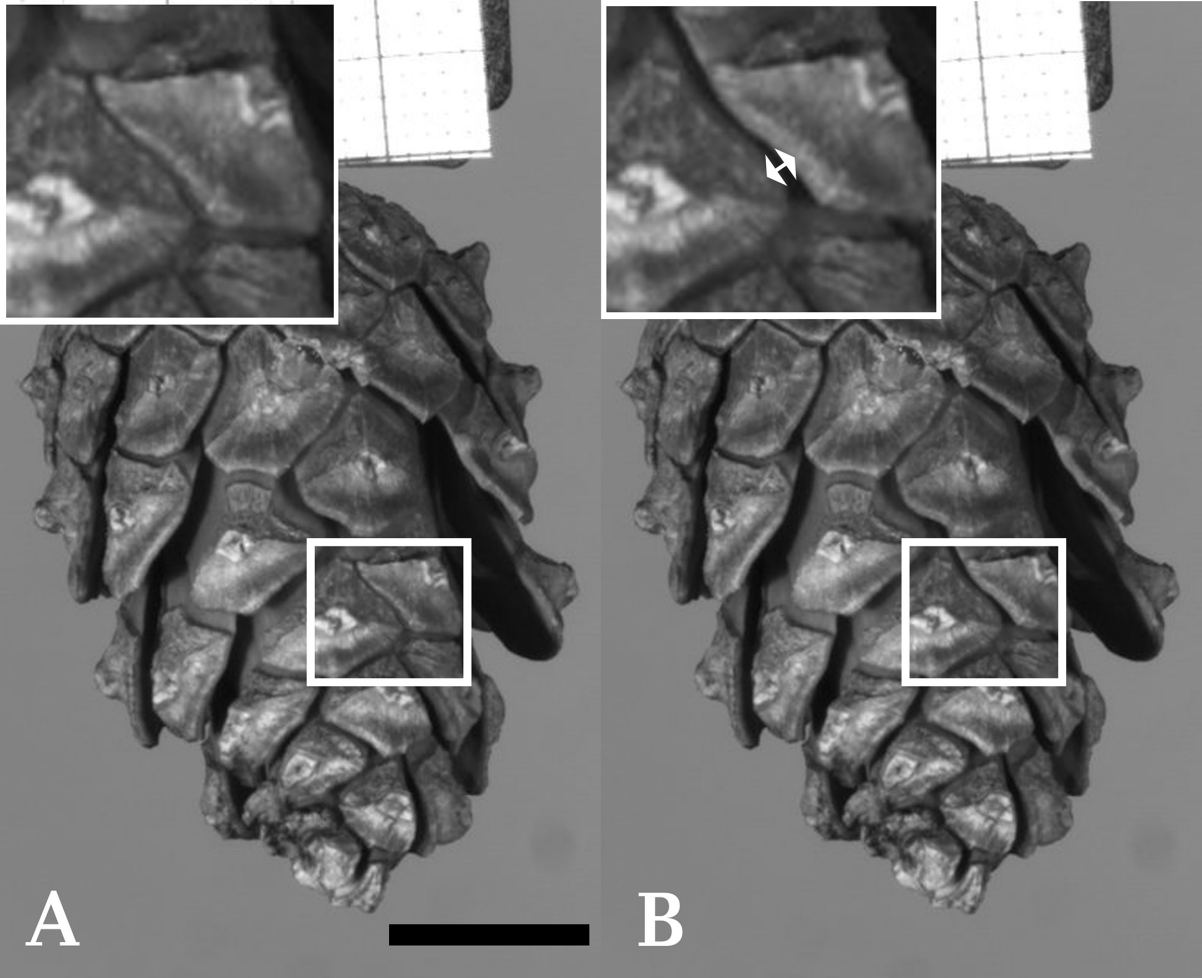
Cracking of an individual seed scale of Scots pine (*Pinus sylvestris*). **(A)** Shows a cone with a highlighted region prior to cracking. Several other scales of the cone have already started to bend. **(B)** Shows the cone after cracking (duration ~2 ms) of a single scale took place (indicated by a double arrow in the enlarged image of the highlighted region). Frames taken from **Supplementary Video S8**. Scale bar = 1 cm.

We captured the initial opening (cracking) of six cones, which resulted in 36 observations of rapid scale movements. Observations varied between one and twelve opening events per cone. Per event, either a single scale moved or several neighboring scales moved simultaneously (on average 3.7 ± 1.5 scales, maximally 7) (**Figure 1** and **Supplementary Videos 1-7**). Under our experimental conditions, the average width of the gaps formed between scales during cracking was 0.2 ± 0.14 mm (min=0.1 mm; max=0.8 mm, n=36). The average scale velocity during cracking was 169 ± 107 mm/s (min=19 mm/s; max=444 mm/s, n=36). During three opening events, vibrations of three individual scales were observed. These vibrations lasted 4.2, 6.5 and again 4.2 ms and were only observed when the respective scale was not in contact with other scales after the initial detachment (**Supplementary Video 8**). One cracking event was immediately followed by a second cracking event. The average duration of cracking events was 1.5 ± 1.0 ms (min=0.6 ms; max=4.2 ms, n=36). Based on the measured average scale velocities during initial cone opening events, accelerations of up to 574 m/s² (59 *g*) were calculated. Average values per opening event are 180 ± 160 m/s² (18 ± 16 *g*). Not all initial scale movements were accompanied by visible or audible cracks. Cone cracking did never lead to an immediate release of seeds in our experiments.

Released seeds (n=8) had an average width of 3.3 (± 0.8) mm (min=2.6 mm; max=4.8 mm) and an average height of 1.9 (± 0.4) mm (min=1.4 mm; max=2.5 mm).

During the secondary opening of the same cones closed after submersion in water over night, no rapid scale movements could be observed (**Supplementary Video 9**).

### 3D digital image correlation analyses of scale deformation and strain during cone opening

Using 3D-DIC, recurring deformation patterns during the drying-induced initial and secondary opening events could not be recognized in the investigated cones. In contrast to the initial opening events, where most positive and negative strains developed relatively rapidly and in an irregular pattern at the border regions between scales, the positive and negative strains during the secondary opening appeared slowly and simultaneously, leading to a smooth cone opening and synchronized movement of scales.

**Figure 2:**
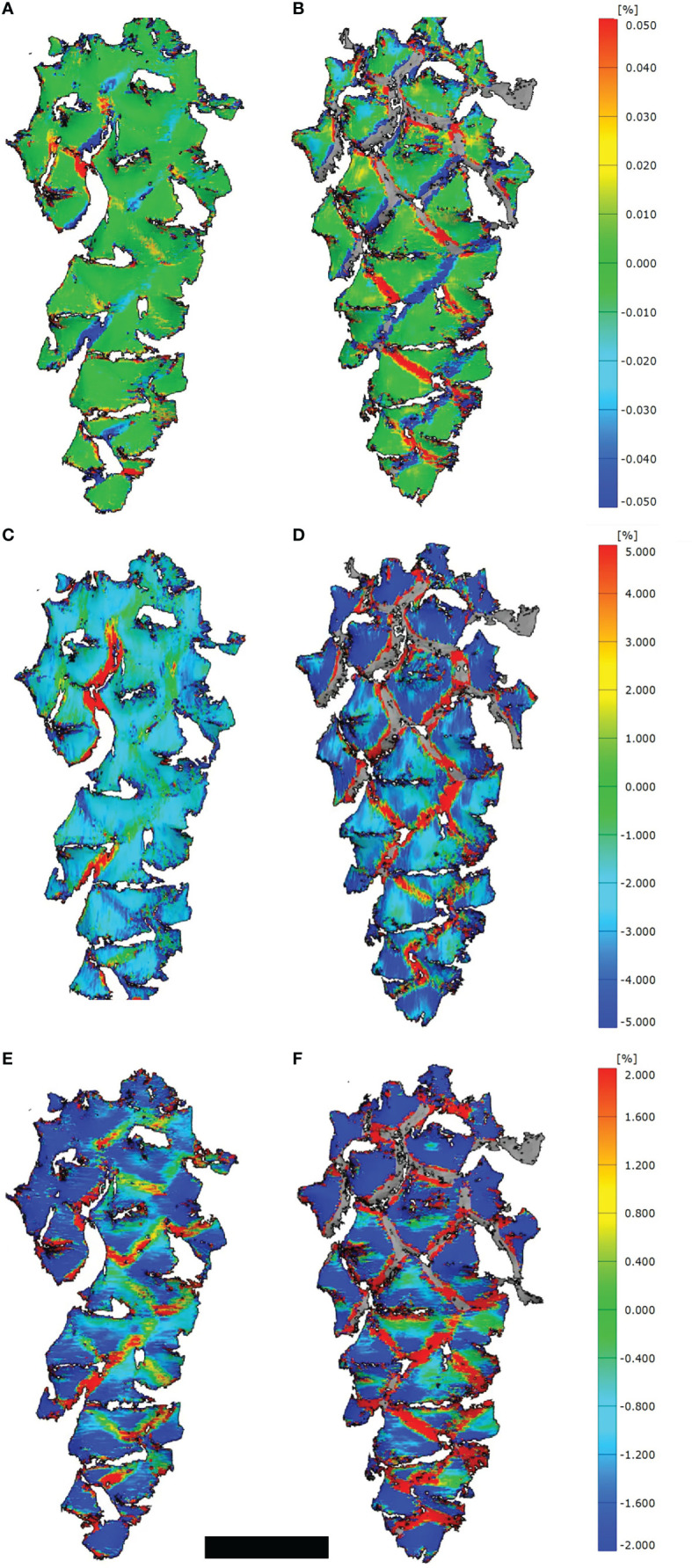
3D-DIC analyses of opening cones. **(A, C, E)** Strains on the cone surfaces during initial opening (t=400 min) and **(B, D, F)** during secondary opening (t=225 min). Given are strains combined for the longitudinal as well as transversal direction **(A, B)**, strains in transversal **(C, D)** and longitudinal direction **(E, F)**. Note the stochastic occurrence and development of strains at the border regions between scales during the initial opening, compared to the regular pattern during the secondary opening. Scale bar = 1 cm.

Despite we measured strains mainly at the border regions between scales (**Figures 2A, B**), some positive and negative strains, especially in longitudinal direction, developed on the umbos as well (**Figures 2E, F**). These strains in transversal direction were generally larger than in longitudinal direction (**Figures 2C, D**).

### Force measurements

The investigated 26 scales from five different cones had an average dry weight of 0.128 ± 0.024 g (min=0.08 g, max=0.171 g) and in the wet state they had an average weight of 0.206 ± 0.04 g (min.: 0.126 g, max.: 0.250 g). During drying, they developed an average force of about 1.17 ± 0.41 N (min=0.46 N; max.: 1.99 N), resulting in average relative forces normed on the respective scale weights of 9.30 ± 3.21 N/g (min=3.56 N/g, max.: 15.1 N/g). The scales were able to store water in the amount of 61.5 ± 13.3% (min=35.6%, max=90.3%) in relation to their dry weight. As scales had contact to the immobile force sensor, they deformed during drying and stayed in the deformed configuration afterwards.

### Temperature of cones during opening

**Figure 3:**
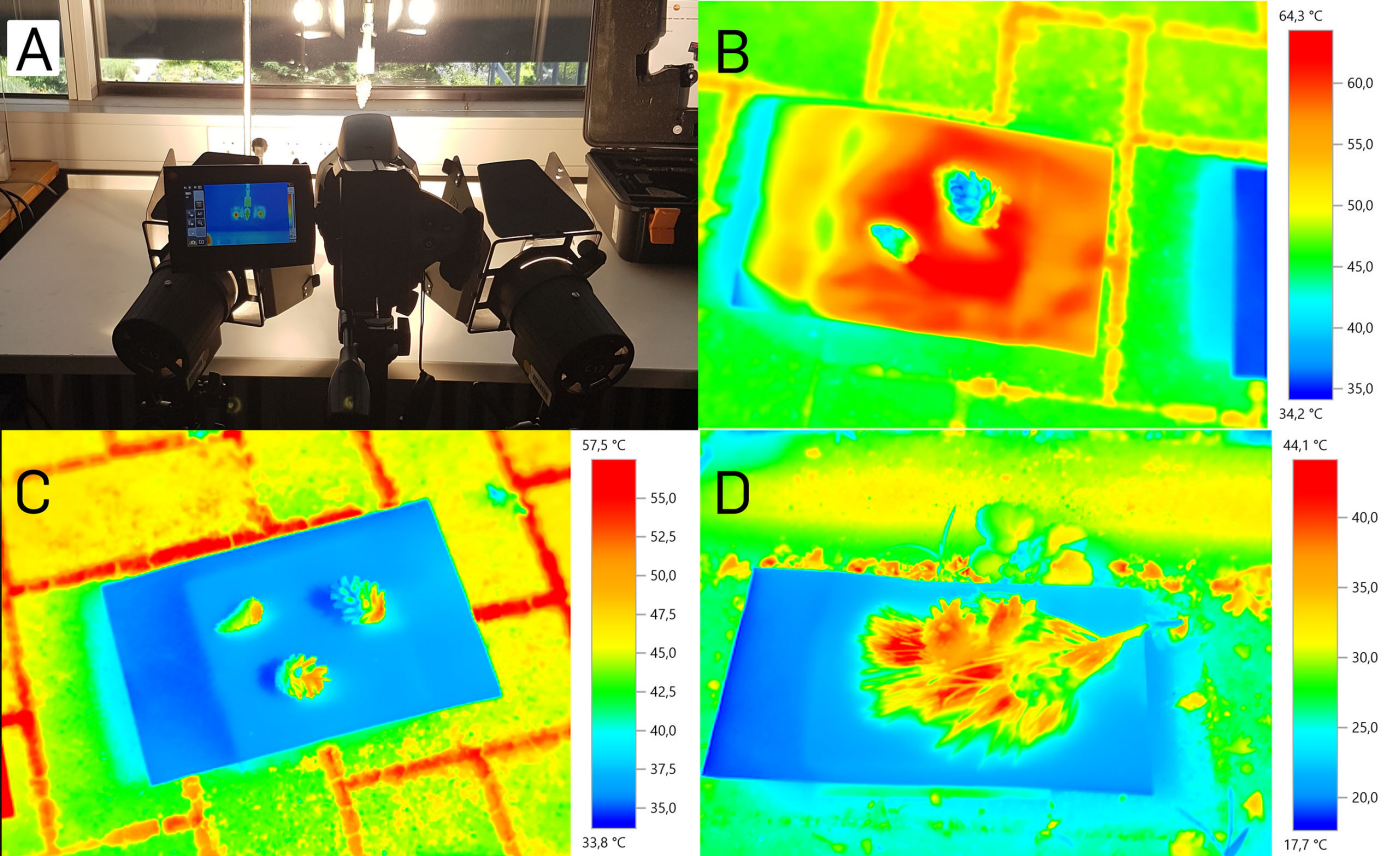
Cone surface temperatures during opening. **(A)** Indoor measurement setup with two artificial light sources. **(B)** Cones in the outdoor experiment on black cardboard. **(C)** Cones in the outdoor experiment on white cardboard. **(D)** Cones in the outdoor experiment on white cardboard surrounded by a pine twig.

We measured the temperature increase as well as the final temperature on the surface of five cones exposed to two artificial light sources (as indicated for the high-speed setup, **Figure 3A**). Within 20 minutes after the start of the experiment, the cones’ surface temperatures increased from 22°C to about 70°C and then stayed constant. The air temperature above the cone was 55°C with 10% relative humidity in this setup (ambient temperature 19°C at 29% relative humidity). Under these conditions, the cones opened within 20-60 minutes.

In the second, more natural setup, we exposed five cones to natural sunlight under the open sky on five days, resembling the conditions under which cones open naturally (**Figures 3B–D**). After 20 minutes of sun exposition, we started the measurements. The cones’ surface temperatures reached a maximal value of 52.7°C, measured with the thermographic camera on a white background. With the infrared thermometer, maximal temperatures of 48°C were measured under the same conditions. On black background, temperatures measured with the thermographic camera reached 53.8°C, measured with the infrared thermometer even 61.6°C. Ambient temperature, and all cardboard background temperatures during both measurements were 29°C at a relative air humidity of 61%. The white background alone reached maximal temperatures of up to 38.2°C (thermographic camera) and 38.4°C (infrared thermometer), respectively. The black background reached temperatures of up to 68°C (thermographic camera) and 63.9°C (infrared thermometer), respectively.

The measurement with the additional *Pinus* twig on white background was conducted at the same place but on another partly sunny, partly cloudy day, with 17°C ambient temperature and 67% relative humidity. A zone of elevated temperature between the needles and around the three cones could be noted, with temperatures of up to 44.1°C (difference to the ambient temperature: 27.1°C). Without the twig, the maximum temperature difference of the cones to the environment was 20.6 °C. On another measurement day with the pine twig where the sun was partly covered with veil clouds, we measured a cone surface temperature of 44.3°C at an ambient temperature of 23.5°C with 52.5% relative humidity (cone surface temperature 20.8°C above ambient). This temperature is even 0.5°C higher than the temperature of cones on black background (43.8°C) measured simultaneously.

During the experiment with cones placed into a fridge, at low temperature (5°C) and low relative air humidity (10-20%), we found that it took cones 5, 5.5 and 7 days to open completely. An increase of temperature afterwards did not lead to a wider opening.

### Resin rheological properties

**Figure 4:**
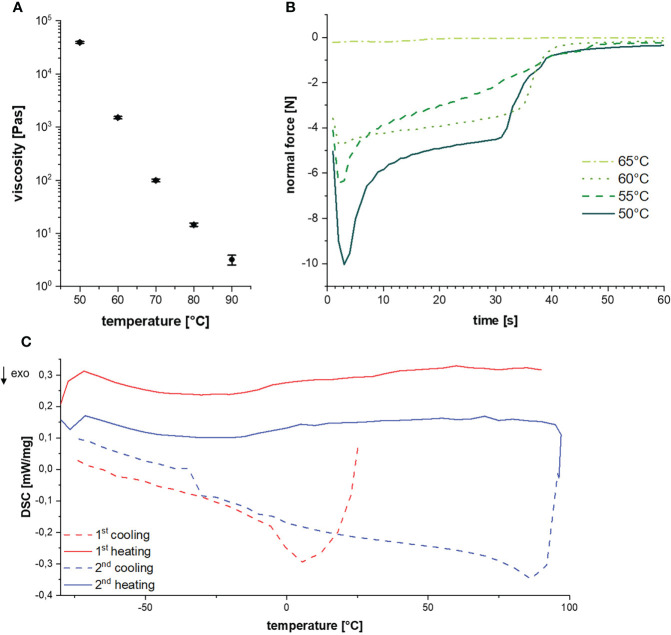
**(A)** Glass-transition and melting temperature during cycled heating and cooling. **(B)** Resin viscosities at different temperatures. **(C)** Diagram of the normal force over time for a lifting speed of 5 µm/s at different temperatures.

The determination of thermal resin properties by DSC showed a glass transition temperature at -16.5°C and a melting temperature between 60 to 70°C (**Figure 4A**). This was confirmed by rheological measurements, which showed that at T < 50°C it was impossible to separate the rheometer metal plates with resin in between. Resin viscosity decreased from 40 kPa at 50°C to 3 Pas at 90°C as shown in **Figure 4B** and **Supplementary Figure 1**. The force required to lift the upper metal plate with 5 µm/s and 1 mm/s, respectively, was also measured as a function of temperature. Lifting the upper plate at 5 µm/s yielded peak forces between -10 and -0.25 N at temperatures between 50 and 65°C, which decreased with increasing temperature (**Figure 4C**). In a more detailed study at temperatures between 55 and 90°C and a lifting speed of 1 mm/s, besides peak force, the adhesive and cohesive strength was also calculated using the integral under the curve. Also, failure time was determined as the value at which the peak force had decreased by 90% (see **Table 1**). All values showed a strong negative temperature dependency.

**Table 1:** Peak forces, adhesive and cohesive strengths, and failure times of Scots pine cone resin under different temperatures regimes at a lifting speed of 1 mm/s.

T [°C]	Tack [N] (Peak force)	Adhesive/Cohesive strength [N/s]	Failure time [s] (Peak force decay by 90%)
55	- 88.04	> 500	> 10
60	- 91.77	59.44	1.33
65	- 73.94	23.97	0.99
70	- 31.51	6.27	0.77
75	- 12.62	4.84	0.83
80	- 7.21	3.00	0.63
90	- 4.43	1.28	0.39

## Discussion

Our results lead us to hypothesize that the initial opening of Scots pine cones is fine-tuned to environmental cues, in particular the interaction of temperature and humidity in combination with the hygroscopicity of seed scales and resin as a functional latch. Previous studies found especially oleoresin, i.e., extracts composed of resin and oils, in cone tissues from various pine species that block cone opening (Kossuth and Biggs, 1981). The melting point of the respective oleoresin was determined at 45-50°C (Critchfield, 1957), which is in general accordance with the results gained in our study.

Pine cones are described in the literature to open and close rather slowly (in the minute or even hours regime) due to changes in environmental humidity (Allen and Wardrop, 1964; Harlow et al., 1964; Dawson et al., 1997; Quan et al., 2021; Eger et al., 2022). Cone cracking, i.e., the initial cone opening, has been described predominantly in the ecological context of serotiny, i.e., adaptations of certain pine species with respect to seed dispersal in fire-prone habitats (Johnson and Gutsell, 1993; Johnson et al., 2003; Wyse et al., 2019b). Our study presents for the first time qualitative and quantitative results on the cracking process, highlighting that the initial scale movement takes place in the millisecond regime. Therefore, pine cones are capable of motions that rank among the fastest plant movements known in terms of movement duration (Skotheim and Mahadevan, 2005; Forterre, 2013; Poppinga et al., 2013). Since no immediate seed release was observed during cone cracking in our experiments, it appears rather unlikely that the rapid scale movements with velocities of up to 444 mm/s and accelerations of up to 180 m/s² function as an explosive release or scatter mechanism as reported from other plant species (Swaine and Beer, 1977; Deegan, 2012; Hofhuis et al., 2016; Poppinga et al., 2019). In the investigation of the initial cone opening, we found that the gap between the scales that arises from the rapid scale movement is too small for seed release, as seeds were at least 1.4-2.6 mm in diameter in our study (compare Novikov and Ivetic, 2018), while the largest found gap was 0.75 mm wide. Therefore, cone cracking did not lead to the forcible ejection and release of seeds in our study.

The observed rapid initial opening of cones sometimes led to audible cracks already described for warm days in spring in pine plantations (Allen and Wardrop, 1964; Wyse et al., 2019a). Subsequent further opening movements of the same cone never were accompanied by sound. The second (and the following) opening events started with the scales situated at the basal part of the cone and transitioned into a simultaneous movement of all scales, whereas, during the initial opening, stochastic cracking events of single or numerous scales were observed. Based on these observations we hypothesize that resin situated between seed scales hinders the hygroscopic scale movement until it ruptures, entailing rapid scale movement, sound generation, and stochastic cracking events. This is further corroborated by the fact that no torn tissue can be observed between the different scales on open cones (cf. **Figure 3**).

We propose the following pine cone initial opening actuation scenario: A mature, closed cone possesses tight resin seals between its individual, wet and bent scales. Under dry conditions in spring, the scales evaporate water and “try” to bend outwards (which would lead to the opening the cone), which is hindered by the resin seals as well as overlaying scales in the Fibonacci series (Lin et al., 2016). Upon suitable conditions regarding temperature and humidity, the forces generated by the hydraulically actuated scales in combination with the temperature-induced alterations of the resin’s rheological properties lead to stochastic seal rupture events. Rupture entails the fast motions of the scales, as observed, driven by the release of stored elastic energy built up by the hygroscopic actuation in concert with some motion clearance gained through the tensioned scales. Within the conceptual framework of “latch-mediated spring actuation” (Longo et al., 2019), the elastic system described here (the closed mature cone) consists of the hygroscopic tissue of the seed scales acting as motor (entailing “loading” of the system), the resin acting as latch (with the temperature-change induced alterations of resin rheological properties entailing triggering/latching), and the deformed scales acting as springs (entailing launching) and as actuated mass. The preloaded scales can most presumably act together mechanically and release other tight connections between scales, leading to the observed movement of up to 7 scales at the same time. However, the synchronous opening of up to 50% of the scales at once as reported from *P. radiata* (Wyse et al., 2019a) could not be observed in our study with Scots pine. Such multi-actuator based composite motile systems are largely unexplored and deserve further investigations especially concerning synergistic mechanical effects between the actuating domains (i.e., the individual scales).

The results from the 3D-DIC analyses support our interpretation. During the investigated initial opening events, strains occurred randomly distributed in between the apophyses, whereas such a stochastic pattern could not be observed during subsequent opening events. Strains in between the apophyses were mainly transversal strains.

The random scale movements during initial opening are contradictory to previous publications that assumed an opening according to the Fibonacci-series in which scales are arranged (Kilmer, 1971; Lin et al., 2016), which in our experiments occurred only from the second opening events onwards. We also observed simultaneous strain developments in between all apophyses in subsequent opening events, which is most probably due to breakage of the resin seals between the scales. We interpret the longitudinal strains to represent an evaporation-induced shrinking of the respective cone, while the strains in transversal direction are higher and mainly represent the opening of the cone scales, based on the breakage of resin seals.

During wetting, the scale dry weights increased on average by about 61.5%, which leads to the typical bending into the closed state of the respective cone (Lin et al., 2016). Some scales nearly doubled their weight (90% increase) during water uptake. Upon desiccation, scales develop an average force of 1.2 N (9 N/g, related to the individual scale weights), with a maximum of 1.99 N (15 N/g), which pushes apart the scales of the cone. These forces measured in *P. sylvestris* are comparable to the forces measured in *P. pinaster*, which is a species with larger cones (Le Duigou and Castro, 2016). Considering that numerous scales on a cone desiccate simultaneously, the resulting forces likely add up, leading to the breakage of the resin seals between the apophyses. If these seals are spatially restricted to form individual, single functional latches, a failure would lead to the observed rapid movement of (individual) scales.

In our temperature measurements we observed that cones absorb sunlight well, as their surface temperatures increased by up to 27°C above air temperature. An earlier study found that solar radiation can heat up cones by 15°C above the environment temperature (Wyse et al., 2019a), which was clearly exceeded in our investigations. That way temperatures of up to 54°C were reached under natural conditions on the cone surfaces tested. Needles of a pine twig presumably act as air cushions, thereby increasing the heating. Dark cardboard background also increased cone surface temperatures. In more detailed experiments with cones placed in a fridge at low humidity, we found how not only temperature, but also humidity interplays in the opening process of *P. sylvestris* cones. We thereby could confirm previous studies (Allen and Wardrop, 1964; Harlow et al., 1964; Dawson et al., 1997). The importance of temperature for the initial opening event could be elucidated in an experiment, in which at temperatures as low as 10°C or beneath (and in a very low relative humidity regime) the opening took up to a week. At temperatures of 50 – 70°C, the whole cone opening occurred within 1 hour, the breaking of all scale connections took even less time. This again emphasizes on the strong regulatory character of temperature on the humidity-driven mechanism. The (sometimes) deviating results for surface temperature measurements with the two used devices are most presumably due to the fact that with the infrared thermometer only a single point on the surface could be measured, while the thermographic camera captured the whole surface, allowing to extract the highest occurring temperature.

Our analyses also confirm temperatures around 50°C that have been measured in previous works on cones of serotinous pines (Perry et al., 1977; Wyse et al., 2019a). Other studies additionally found a strong correlation of Sharav events (days of hot and dry weather in the Eastern Mediterranean region) with seed release in *Pinus halepensis* (Nathan et al., 1999), which is a serotinous species as well. Presumably, the heat-triggered initial opening is even more fine-tuned in such serotinous species as compared to the here investigated Scots pine.

**Figure 5:**
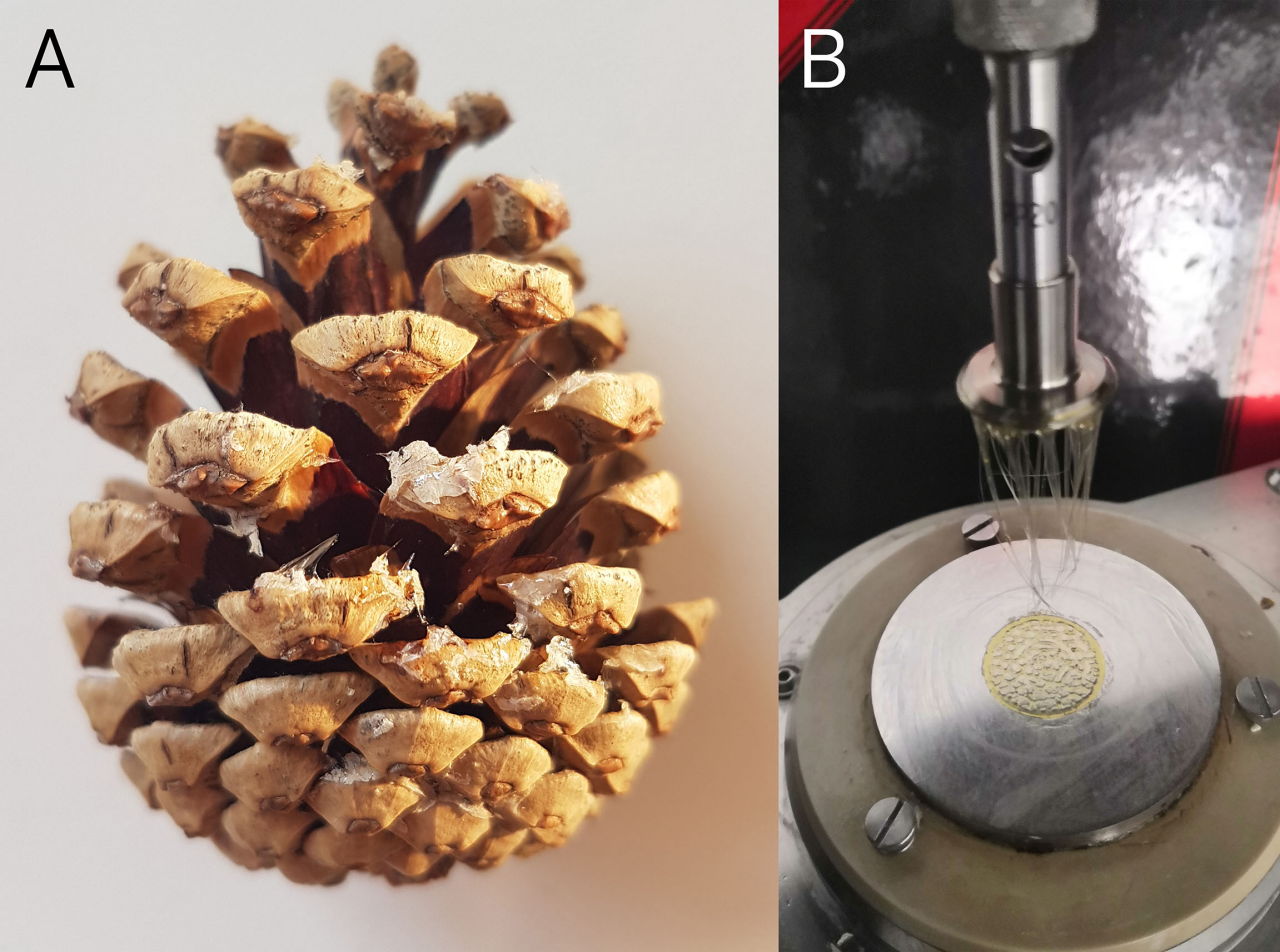
Resin threads. **(A)** Naturally occurring threads of resin between the scales of a *P. nigra* cone. **(B)** Experimental resin threads produced during rheological measurements by pulling apart two metal plates glued by resin from *P. sylvestris* cones.

Regarding the resin rheological properties, we observed extremely high forces required for tearing apart rapidly two metal plates glued with resin. Only from approximately 50°C onwards the resin is liquified enough and it was possible to pull apart the glued plates in our test setup. At the investigated movement speed of 5 µm/s, temporal forces between 4.7 and 10 N are required to pull the plates apart. Since the measurement setup consisted of metal plates instead of organic material, and since the amount of glue and the area it covers is different to the situation in the natural pine cone, the required forces cannot be directly compared to the measured forces pine cone scales developed. However, the results gained allow for an approximate comparison, in which it can be seen that the forces required in the test setup and the forces generated by the scales are in the same order of magnitude. With permanent tension, as in the case of the movement of the individual scales, and higher temperatures the resin presumably begins to flow (i.e., becomes less viscous) slowly over time, so that lower forces are required in this case and the force of 1.99 N of an individual scale is sufficient to overcome the adhesive force of the resin. Apparently, the resin gives way in a cohesive manner, as pulling threads were observed in nature as well as experiment (**Figure 5**). Therefore, we assume that high temperatures (~50°C) facilitate the opening of *P. sylvestris* cones. High humidity has no influence on rheological properties of resin, as it is insoluble in water, which has therefore no potential to liquify it.

## Conclusions

Based on our results, we are able to propose a holistic theory on pine cone initial opening for the Scots pine (*P. sylvestris*). We suggest the resin in between the scales respectively at the apophyses to glue the scales together. That way the cone and especially the seeds are protected from bacteria and/or fungi, herbivores, and potentially also from fires as in serotinous species (Campbell, 1955; Carlisle and Brown, 1968; Keeley, 2012). Furthermore, resin is located within the scales in resin ducts (Allen and Wardrop, 1964; Roy, 1966; Lin et al., 2002; Bae and Kim, 2020). We assume that at colder temperatures bending is slowed down by cold and solid resin, but is not completely prevented, as under accumulating stress the resin may start to flow over time under constant tension and therefore allow the cone to open. The bending force elicited under low humidity may, therefore, be counteracted by the stiff resin. On the contrary, at high temperatures, water loss is sped up and the resin is liquified, notwithstanding the bending movement of the scale. That way, temperature and humidity must be in a certain range to facilitate opening within a certain time frame. We assume that the double locking of the scales by these environmental factors may provide an ecologically optimized seed release (Nathan et al., 1999), however, this remains to be tested experimentally.

In a low temperature regime, the cone opening due to low humidity, as tested, is too slow to be finished within one day, which may be speculated to prohibit seed release during cold and dry winter days. During nighttime temperature decrease, which is typically accompanied by an increase in humidity, the opening process would be set to its start condition. On warm but humid days, the opening of cones is not initiated, as the desiccation-induced movement cannot take place. That prohibits the release of seeds on warm but rainy days, which would severely reduce dispersal distance, as seeds are pushed to the ground by precipitation (Nathan et al., 1999; Wyse et al., 2019b). Only on days where humidity is low and temperatures are quite high, both criteria for seed release are met. As seeds and cones of Scots pine ripen in autumn/winter of the second year under natural circumstances, these conditions are met on warm and dry days after winter (Carlisle and Brown, 1968). During these days long-distance dispersal of seeds is likely, and the onset of the vegetation period allows a potentially successful start for the new seedlings. We therefore assume the seed release of *P. sylvestris* to be tailored to allow for good seed dispersal and a successful seedling establishment while at the same time it enables maximized protection against threats to the day of seed release. Future investigations could be extended to include the opening process of cones from other conifer genera (e.g., Aniszewska, 2010; Tulska et al., 2021).

## Data availability statement

The original contributions presented in the study are included in the article/**Supplementary Material**. Further inquiries can be directed to the corresponding authors.

## Author contributions

MH, SP and HB designed the experiments. MH and SP discussed early stages of this study. MH and HB conducted the experiments. MH, SP and HB analyzed the data. MH wrote the first draft of the manuscript. SP and TS supervised the study and acquired the funding. TS contributed expertise and laboratory facilities. All authors have read and discussed the manuscript and agreed to the final version of the manuscript.

## Funding

MH and TS would like to thank the MWK-Baden-Württemberg for financial support of the project “Bio-inspirierte elastische Materialsysteme und Verbund-Komponenten für nachhaltiges Bauen im 21ten Jahrhundert (BioElast)”. HB, TS and SP acknowledge the financial support by BASF SE, Ludwigshafen, Germany, and the Ministry of Science Research and Arts of the State of Baden-Württemberg, Germany, who supported this research within the frame of the Academic Research Alliance JONAS (“Joint Research Network on Advanced Materials and Systems”) established jointly with BASF SE and the University of Freiburg, Germany. TS and SP were additionally funded by the Deutsche Forschungsgemeinschaft (DFG, German Research Foundation) under Germany’s Excellence Strategy – EXC-2193/1 – 390951807. We acknowledge support by the Deutsche Forschungsgemeinschaft (DFG – German Research Foundation) and the Open Access Publishing Fund of Technical University of Darmstadt.

## Acknowledgments

The authors gratefully acknowledge Andreas Warmbold (University of Freiburg, Freiburg Materials Research Center) for his help with the DSC measurements.

## Conflict of interest

The authors declare that this study received funding from BASF SE, Ludwigshafen, Germany. The funder was not involved in the study design, collection, analysis, interpretation of data, the writing of this article, or the decision to submit it for publication.

## Publisher’s note

All claims expressed in this article are solely those of the authors and do not necessarily represent those of their affiliated organizations, or those of the publisher, the editors and the reviewers. Any product that may be evaluated in this article, or claim that may be made by its manufacturer, is not guaranteed or endorsed by the publisher.
