# Supplementary material for: The cracking of Scots pine (Pinus sylvestris) cones
Source: Front Plant Sci. 2022 Oct 18;13:982756. doi: 10.3389/fpls.2022.982756 (PMC9623100; doi:10.3389/fpls.2022.982756)
Supplement: Supplementary file 1 [file DataSheet_1.pdf]

SUPPLEMENTARY MATERIAL FOR

The cracking of Scots pine (*Pinus sylvestris*) cones

Martin Horstmann[1, 2*], Hannah Buchheit[3], Thomas Speck[2, 4] and Simon Poppinga[5*]

[1]Ruhr University Bochum, Bochum, Germany

[2]Plant Biomechanics Group, Botanic Garden, University of Freiburg, Freiburg, Germany

[3]Freiburg Materials Research Center and Institute for Macromolecular Chemistry, University of Freiburg, Freiburg, Germany

[4]Cluster of Excellence livMatS, University of Freiburg, Freiburg, Germany

[5] Botanical Garden, Department of Biology, Technical University of Darmstadt, Germany

Supplementary material descriptions

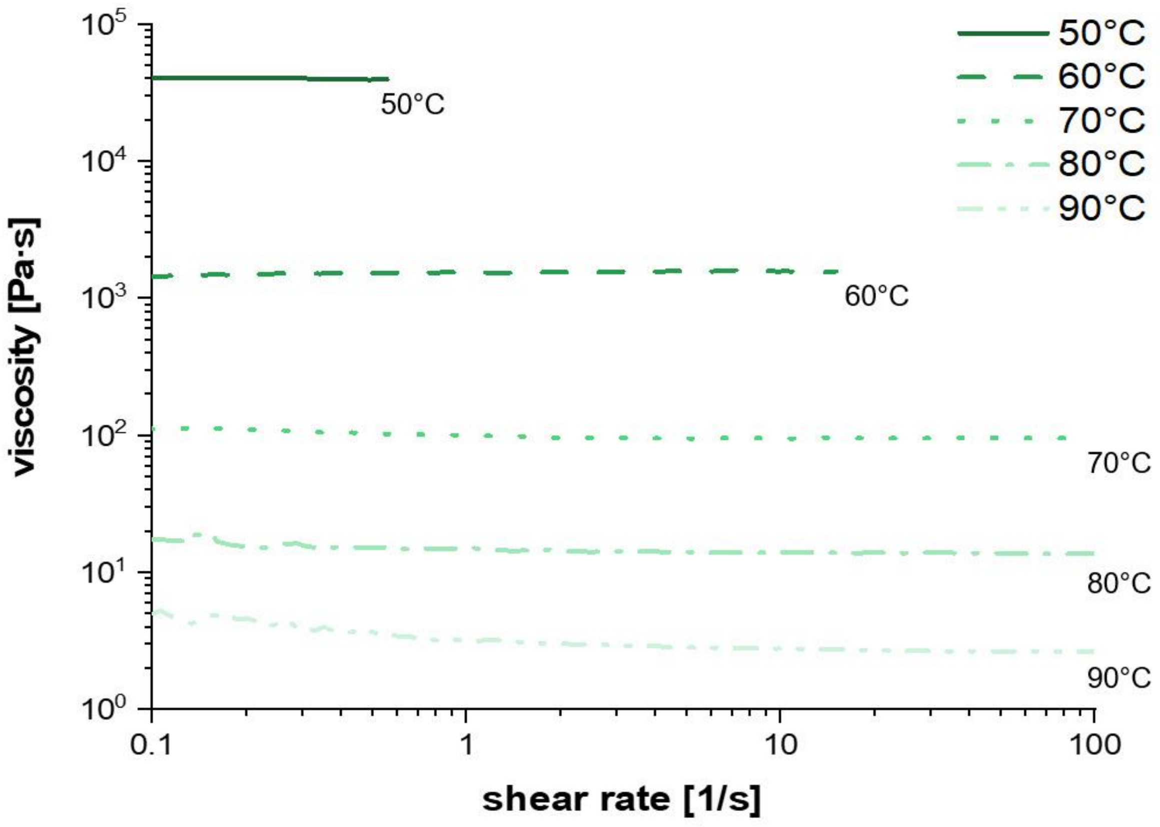

S-Figure 1: **Rheometric analysis.** Graph showing shear rate-independent viscosity of resin from *P. sylvestris* at different temperatures.

S-Video 1-7: **Rapid scale movement.** Rapid initial cone opening with 1-7 scales involved simultaneously.

S-Video 8: **Rapid cone opening.** In the highspeed video footage the rapid scale movements during the first cone opening can be observed, including a free-swinging movement.

S-Video 9: **Comparison of initial and secondary opening.** This compilation of a first and secondary opening shows the random and abrupt movements of scales during the initial cone opening. In the second part of the video, the secondary opening of the same cone is visible, which starts synchronized in the basal part of the cone and continues to the tip of the cone.
